# Associations between Social Support and Patient-Reported Outcomes in Patients Receiving Hemodialysis: Results from the TACcare Study

**DOI:** 10.34067/KID.0000000000000456

**Published:** 2024-05-03

**Authors:** Sarah J. Erickson, Jonathan G. Yabes, Zhuoheng Han, Maria-Eleni Roumelioti, Bruce L. Rollman, Steven D. Weisbord, Jennifer L. Steel, Mark L. Unruh, Manisha Jhamb

**Affiliations:** 1 Department of Psychology, University of New Mexico, Albuquerque, New Mexico; 2 Division of General Internal Medicine, Department of Medicine and Biostatistics, Center for Research on Heath Care Data Center, University of Pittsburgh, Pittsburgh, Pennsylvania; 3 Renal-Electrolyte Division, Department of Medicine, University of Pittsburgh School of Medicine, Pittsburgh, Pennsylvania; 4 Division of Nephrology, Department of Internal Medicine, University of New Mexico School of Medicine Albuquerque, New Mexico; 5 Division of General Internal Medicine, Center for Behavioral Health, Media, and Technology, University of Pittsburgh, Pittsburgh, Pennsylvania; 6 Renal Section, Center for Health Equity Research and Promotion, VA Pittsburgh Healthcare System, Pittsburgh, Pennsylvania; 7 Department of Surgery, Psychiatry and Psychology, University of Pittsburgh, Pittsburgh, Pennsylvania

**Keywords:** dialysis, ESKD, hemodialysis, patient self-assessment, quality of life

## Abstract

**Key Points:**

- Mean baseline levels of perceived social support (Multidimensional Scale of Perceived Social Support) were comparable with other chronically ill populations.
- Higher Multidimensional Scale of Perceived Social Support scores were correlated with lower levels of fatigue, pain, depressive symptoms, anxiety, better sleep quality, and health-related quality of life (Short Form-12 Mental Component Score).
- Moderation analyses revealed male sex and non-Hispanic ethnicity resulted in stronger positive associations of perceived social support with Short Form-12 Mental Component Score.

**Background:**

Patients with ESKD experience high symptom burden, which has been associated with a negative effect on their interpersonal relationships. However, there is limited research exploring associations of social support and patient-reported outcomes among patients receiving hemodialysis.

**Methods:**

This study is a secondary, cross-sectional analyses of the sociodemographic and clinical correlates of perceived social support (Multidimensional Scale of Perceived Social Support [MSPSS]) at baseline. The study examined the extent to which perceived social support is associated with pain, depression, fatigue, anxiety, sleep, and health-related quality of life (Short Form-12 [SF-12] Mental Component Score [MCS] and Physical Component Score.

**Results:**

Of the 160 randomized patients, the mean (SD) age was 58±14 years; years on dialysis was 4.1±4.2; 45% were female; 29% Black, 13% American Indian, and 18% Hispanic; 88% had at least high school education; and 27% were married. Mean baseline levels of perceived social support were comparable with other chronically ill populations. At least high school education (*P* = 0.04) and being married (*P* = 0.05) were associated with higher total MSPSS scores. Higher MSPSS scores were correlated with lower levels of fatigue (*r*=0.21, *P* = 0.008; higher fatigue scores signify lower fatigue), pain (*r*=−0.17, *P* = 0.03), depressive symptoms (*r*=−0.26, *P* < 0.001), anxiety (*r*=−0.23, *P* = 0.004), better sleep quality (*r*=−0.32, *P* < 0.001), and SF-12 MCS (*r*=0.26, *P* < 0.001). Moderation analyses revealed male sex and non-Hispanic ethnicity resulted in stronger positive associations of perceived social support with SF-12 MCS.

**Conclusions:**

The level of perceived social support observed among patients receiving thrice-weekly hemodialysis in Technology Assisted Stepped Collaborative Care was similar to those observed in other chronic conditions. Because of the associations between perceived social support and patient-reported outcomes, particularly psychosocial and behavioral health outcomes, targeting social support appears to be warranted among patients receiving hemodialysis.

**Clinical Trial registration number:**

ClinicalTrials.gov NCT03440853.

## Visual Abstract

**Figure:**
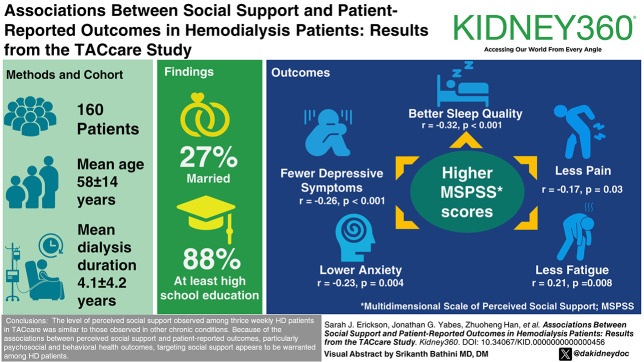

## Introduction

ESKD is associated with high symptom burden, poor health-related quality of life (HRQoL), and increased risk of mortality.^1–4^ HRQoL refers to the multidimensional subjective assessment of the disease's effect across physical, psychological, and social functioning domains.^5^ Because of this high symptom burden and the demands of thrice-weekly hemodialysis, patients receiving hemodialysis experience limitations in their daily lives, including social isolation and a negative effect on their relationships with family, friends, and significant others.^6^ However, there is limited research exploring associations of social support with patient-reported outcomes among patients receiving hemodialysis.

A review of studies addressing social support among patients with CKD found that social support may positively affect a range of outcomes, including improved survival rates, through a variety of hypothesized mechanisms, including decreased depressive symptoms, improved HRQoL, greater access to health care, increased medical adherence, and/or effects on the immune system.^7^ Of the studies including sources of social support (family, friends, significant others), findings suggest that as social support increases among patients receiving hemodialysis, they report greater pleasure, fewer depressive symptoms, less illness burden, and increased coping with fatigue and stress.^8,9^

A more recent study investigating the relationship between fatigue and social support in patients receiving hemodialysis found a negative association between support from family, friends, and a significant other and overall support and fatigue severity.^10^ In fact, support from family was rated the highest of support sources while support from friends was found to be uniquely predictive of HRQoL among patients receiving hemodialysis.^11^ Another study found a strong correlation between HRQoL and psychosocial factors, including social support, but a weaker association with medical factors, such as contributing factors of ESKD, duration of hemodialysis, and comorbidities among patients receiving hemodialysis.^12^ Furthermore, social support from family, friends, and significant others has been shown to be inversely associated with anxiety and depression among patients receiving hemodialysis.^13^ In addition, social support interventions have been shown to improve the HRQoL of patients with ESKD.^14^

Most extant studies investigating these associations have been from other countries, and the limited studies from the United States often did not include diverse samples. Different countries and cultures may have different conceptualizations of and expectations about social support,^15^ with some preliminary evidence that social support may have culturally based differential associations with disease-related characteristics among patients receiving hemodialysis.^16^ This current study leverages a moderate-large and diverse population of patients receiving hemodialysis who were included in a trial testing the efficacy of Technology Assisted Stepped Collaborative Care (TACcare), a stepped collaborative care intervention, to examine sociodemographic and disease-related characteristics that affect perceived social support and the association with patient-reported outcomes assessed at baseline. The role of culture (with race and ethnicity as proxies), as well as other potential moderators (sex, age), was examined with regard to the association between social support and a range of patient-reported outcomes.

## Methods

### Participants

Participants who completed baseline data and enrolled in the TACcare trial were included in this analysis. The study intentionally recruited a representative sample of participants with exclusion criteria to select a group of participants who screened positive for depressive symptoms, pain, and fatigue and participated in a stepped collaborative care intervention. Inclusion criteria included age 18 years and English-speaking patients undergoing thrice-weekly maintenance hemodialysis for >3 months and would require dialysis for at least 6 months. A total of 101 of 896 patients with ESKD (11.3%) assessed for study eligibility were ineligible because of limited English proficiency. The study exclusion criteria included thought disorder, delusions, or active suicidal intent; active substance abuse; too ill or cognitively impaired to participate on the basis of clinicians' judgment; anticipated life expectancy of <1 year; and scheduled for a living donor kidney transplant or relocating to another dialysis unit within 6 months. Details of the study design have been reported elsewhere.^17^ This study was approved by the University of Pittsburgh's Institutional Review Board and the University of New Mexico's Human Research Review Committee. Participants were treated in accordance with the Declaration of Helsinki.

### Measures

The following measures were obtained at baseline: *Multidimensional Scale of Perceived Social Support (MSPSS)*^18^ is a 12-item self-report questionnaire to measure an individual's perceived level of social support from family, friends, and significant others. Each item is scored on a scale from 1 (very strongly disagree) to 7 (very strongly agree). The MSPSS has three subscales, with four items each assessing perceived support from family (*e.g*., “I get the emotional help and support I need from my family”), friends (*e.g*., “My friends really try to help me”), and a significant other (*e.g*., “There is a special person in my life who cares about my feelings”). Subscale and total scale scores are calculated by summing items. Higher scores indicate greater perceived social support. The MSPSS has demonstrated good reliability and validity across populations, including those with ESKD ^19^ and other chronic illnesses.^20^

Additional baseline variables included fatigue (Functional Assessment of Chronic Illness Therapy Fatigue^21^ score, range 0–52, higher score indicates less fatigue), pain severity (Brief Pain Inventory Short Form^22^ average pain severity item score, range 0–10, higher score indicates worse pain), depression (Beck Depression Inventory-II^23^ score, range 0–63, higher score indicates more severe depressive symptoms), sleep quality (Pittsburgh Sleep Quality Index^24^ scores range from 0 to 57, with higher scores indicating more sleep disturbances), anxiety (Generalized Anxiety Disorder-7,^25^ range 0–21, with higher scores indicating more severe anxiety symptoms), physical activity (Physical Activity Scale for Elderly,^26^ range 0–793, measuring leisure, household, and occupational activity with higher scores indicating greater physical activity), HRQOL (Medical Outcomes Study Short Form-12 [SF-12],^27^ using norm-based scoring [mean±SD, 50±10] with higher scores indicating better self-reported health, yielding a physical component score [SF-12 PCS] and a mental component score [SF-12 MCS]), and adult global health (NIH Patient-Reported Outcomes Measurement Information System [PROMIS]^28^ with eight profile domains: fatigue, pain intensity, pain interference, physical function, sleep disturbance, anxiety, depression, and ability to participate in social roles and activities, using T scores [mean±SD, 50±10], with higher scores indicating greater severity).

### Statistical Analyses

Baseline data from all participants in the TACcare trial were included in the analyses. Means with standard deviations and counts and percentages were used to descriptively summarize patient characteristics and patient-reported outcomes by tertiles of MSPSS, and *P* values were derived from ANOVA or chi-square tests. To assess the bivariate associations between social support and patient characteristics, we calculated the mean MSPSS and associated 95% confidence intervals stratified by baseline characteristic subgroups. We performed Pearson correlation analyses to quantify the bivariate associations between social support and various patient-reported outcomes. We used multivariable linear regression models to assess adjusted associations between social support (primary covariate) and SF-12 PCS and SF-12 MCS (outcomes) by sequential addition of patient characteristics in the model. Model 0 (unadjusted) only included total MSPSS (unadjusted); in model 1, we adjusted for age, sex, race, and ethnicity; model 2 (full model) additionally included Charlson Comorbidity Index (CCI). These covariates were included on the basis of clinical rather than statistical reasons. We added interaction terms to model 2 to determine whether sex or ethnicity moderated the association between social support and SF-12 MCS. All analyses were performed using R version 4.3.1,^29^ and results with a *P* value ≤ 0.05 were considered statistically significant.

## Results

**Figure 1:**
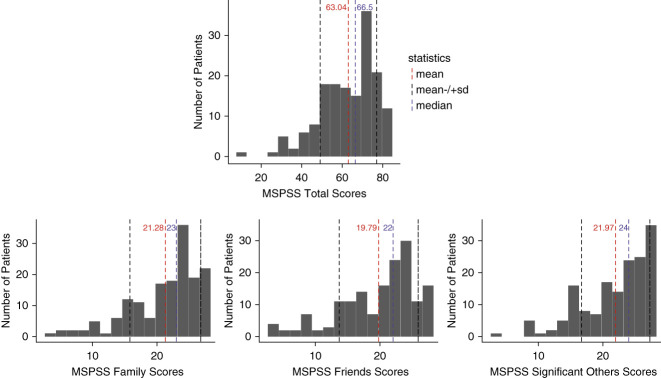
Baseline levels of perceived social support.

**Figure 2:**
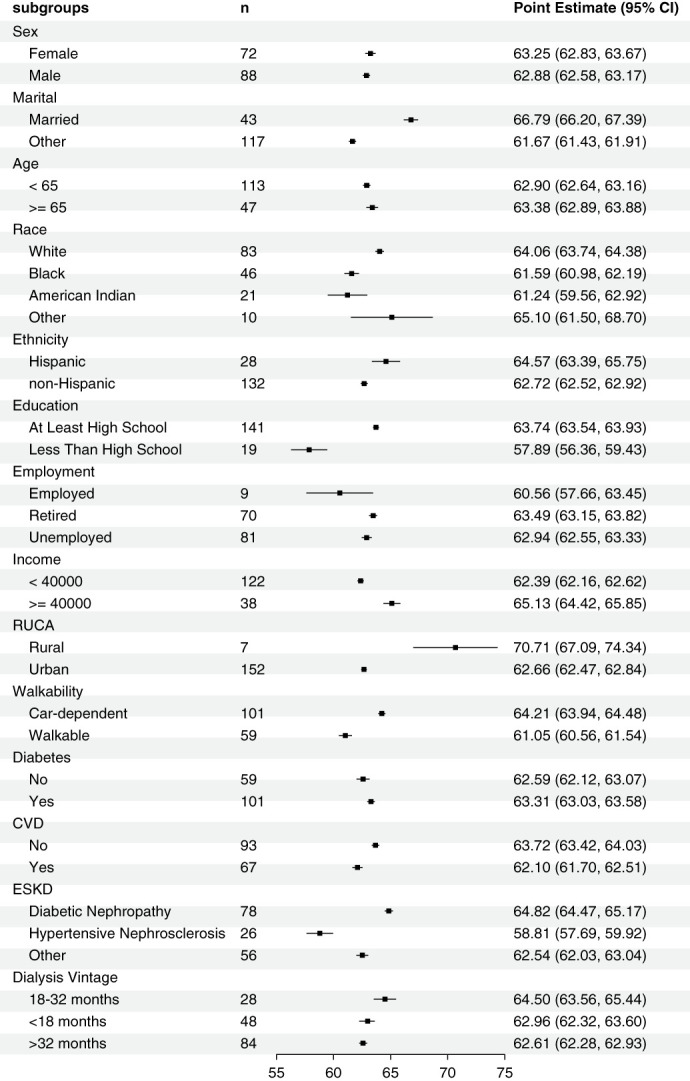
**MSPSS total with respect to sociodemographic and disease-related characteristics.** CI, confidence interval; CVD, cardiovascular disease; MSPSS, Multidimensional Scale of Perceived Social Support; RUCA, rural–urban commuting area code.

Of the 160 randomized patients included in this study, the mean (SD) age was 58±14 years; 45% were female; 29% were Black, 13% American Indian, and 18% Hispanic; 88% had at least a high school education; 27% were married and were on dialysis for 4.13±4.18 years (Table 1). Mean baseline levels of MSPSS scores from family, friends, a significant other, and total scores were 21.3 (SD=5.5), 19.8 (SD=6.1), 22.0 (SD=5.3), and 63.0 (SD=14.0), respectively (Figure 1). Being married (mean MSPSS for married: 66.8 versus other: 61.7; *P* = 0.05) and completing high school or above (mean MSPSS for at least high school: 63.7 versus less than high school: 57.9; *P* = 0.04) were associated with significantly higher total MSPSS scores, but no disease-related characteristics were associated with total MSPSS scores (Figure 2 and Supplemental Table 1).

**Table 1:** Baseline characteristics of patients randomized in the Technology Assisted Stepped Collaborative Care trial by Multidimensional Scale of Perceived Social Support tertile

Characteristics	All (*N*=160)	MSPSS T1 (*N*=54)	MSPSS T2 (*N*=61)	MSPSS T3 (*N*=45)	*P* Value
Age, mean (SD)	57.87 (13.82)	57.77 (12.32)	57.90 (15.39)	57.94 (13.60)	>0.9
Female, *n* (%)	72 (45)	26 (48)	22 (36)	24 (53)	0.2
Married, *n* (%)	43 (27)	8 (15)	21 (34)	14 (31)	0.046
**Race** **, *n* (%)**					0.5
White	83 (52)	27 (50)	29 (48)	27 (60)	
Black	46 (29)	17 (31)	21 (34)	8 (18)	
American Indian	21 (13)	8 (15)	7 (11)	6 (13)	
Other/missing	10 (6.2)	2 (3.7)	4 (6.6)	4 (8.9)	
Hispanic, *n* (%)	28 (18)	7 (13)	11 (18)	10 (22)	0.5
Education (HS or greater), *n* (%)	141 (88)	44 (81)	53 (87)	44 (98)	0.041
Employed, *n* (%)	9 (5.6)	5 (9.3)	2 (3.3)	2 (4.4)	0.6
Tobacco use ever, *n* (%)	86 (54)	31 (57)	34 (56)	21 (47)	0.5
Current alcohol use, *n* (%)	25 (16)	13 (24)	8 (13)	4 (8.9)	0.092
Household income (<$40,000/yr), *n* (%)	122 (76)	42 (78)	47 (77)	33 (73)	0.9
Diabetes, *n* (%)	101 (63)	35 (65)	37 (61)	29 (64)	0.9
Cardiovascular disease, *n* (%)	67 (42)	25 (46)	27 (44)	15 (33)	0.4
CCI	4.77 (1.77)	4.72 (1.60)	4.75 (1.76)	4.84 (2.02)	>0.9
**Etiology of ESKD** **, *n* (%)**					0.5
Diabetic nephropathy	78 (49)	24 (44)	28 (46)	26 (58)	
Hypertensive nephrosclerosis	26 (16)	11 (20)	11 (18)	4 (8.9)	
Other	56 (35)	19 (35)	22 (36)	15 (33)	
Dialysis vintage, yr, mean (SD)	4.13 (4.18)	4.51 (4.48)	4.09 (4.07)	3.72 (4.02)	0.4
Psychotherapy within 6 mo prior to study, *n* (%)	19 (12)	9 (17)	5 (8.2)	5 (11)	0.4
Antidepressant use, *n* (%)	58 (36)	21 (39)	18 (30)	19 (42)	0.4
Opioid use, *n* (%)	48 (30)	16 (30)	15 (25)	17 (38)	0.4
FACIT-fatigue score, mean (SD)	28.29 (10.98)	24.44 (11.73)	30.67 (9.50)	29.67 (10.90)	0.012
Fatigue (FACIT-F ≤44), *n* (%)	152 (95)	53 (98)	57 (93)	42 (93)	0.5
BPI pain score, mean (SD)	3.66 (3.25)	4.44 (3.43)	3.66 (3.09)	2.71 (3.05)	0.029
Mod.-severe pain (BPI ≥5), *n* (%)	74 (46)	31 (57)	26 (43)	17 (38)	0.11
BDI depression score, mean (SD)	15.51 (8.57)	18.35 (10.11)	13.67 (7.26)	14.60 (7.41)	0.023
Depression (BDI ≥16), *n* (%)	69 (43)	32 (59)	22 (36)	15 (33)	0.013
PASE physical activity, mean (SD)	229.63 (53.98)	219.83 (45.09)	230.87 (56.15)	239.69 (59.65)	0.2
GAD-7, mean (SD)	6.00 (4.80)	7.41 (5.07)	4.98 (4.55)	5.69 (4.49)	0.023
PSQI sleep score, mean (SD)	8.93 (3.40)	10.17 (3.77)	8.28 (3.26)	8.33 (2.71)	0.017
Sleep (PSQI ≥5), *n* (%)	145 (91)	51 (94)	54 (89)	40 (89)	0.5
SF-12 PCS, mean (SD)	34.80 (8.29)	33.52 (8.23)	35.88 (8.11)	34.89 (8.56)	0.4
SF-12 MCS, mean (SD)	39.68 (8.75)	36.46 (9.01)	41.36 (7.42)	41.29 (9.18)	0.003
PROMIS anxiety, mean (SD)	53.35 (9.13)	55.49 (8.58)	52.30 (9.32)	52.21 (9.24)	0.13
PROMIS depression, mean (SD)	53.36 (8.98)	57.42 (8.69)	52.22 (8.12)	50.04 (8.79)	<0.001
PROMIS fatigue, mean (SD)	57.92 (8.78)	60.90 (8.45)	55.74 (7.96)	57.25 (9.37)	0.008
PROMIS pain interference, mean (SD)	59.73 (9.49)	62.65 (9.68)	58.61 (9.54)	57.77 (8.49)	0.016
PROMIS physical function, mean (SD)	38.08 (7.96)	38.64 (7.19)	37.74 (8.68)	37.85 (7.97)	0.5
PROMIS sleep disturbance, mean (SD)	52.03 (3.76)	52.45 (4.25)	52.08 (3.30)	51.46 (3.73)	0.2
PROMIS social roles, mean (SD)	45.79 (8.39)	48.82 (8.08)	44.20 (8.10)	44.32 (8.32)	0.002

BDI, Beck Depression Inventory; BPI, Brief Pain Inventory-Short Form; CCI, Charlson Comorbidity Index; FACIT-F, Functional Assessment of Chronic Illness Therapy Fatigue; GAD-7, Generalized Anxiety Disorder-7; MCS, Mental Component Score; MSPSS, Multidimensional Scale of Perceived Social Support, MSPSS T1 (first tertile: 12–57), MSPSS T2 (second tertile: 58–72), MSPSS T3 (third tertile: 73–84), PASE, Physical Activity Scale for Elderly; PCS, Physical Component Score; PROMIS, NIH Patient-Reported Outcomes Measurement Information System; PSQI, Pittsburgh Sleep Quality Index, Medical Outcomes Study Short Form-12 (SF-12) Physical Component Score (SF-12 PCS) and Mental Component Score (SF-12 MHS).

**Figure 3:**
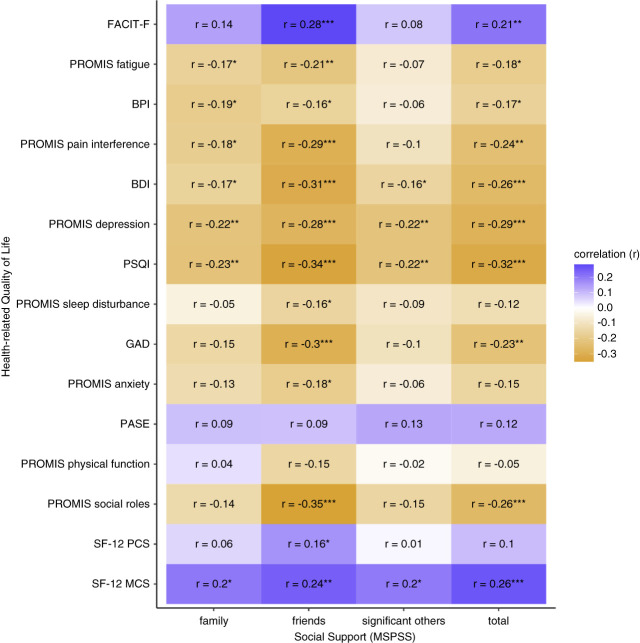
**Correlations between HRQoL outcomes and perceived social support.** *P value < 0.05; **P value < 0.01; ***P value < 0.001. BDI, Beck Depression Inventory; BPI, Brief Pain Inventory-Short Form; GAD-7, Generalized Anxiety Disorder-7; FACIT-F, Functional Assessment of Chronic Illness Therapy Fatigue; HRQoL, health-related quality of life; PROMIS, NIH Patient-Reported Outcomes Measurement Information System; PSQI, Pittsburgh Sleep Quality Index; PASE, Physical Activity Scale for Elderly, Medical Outcomes Study Short Form-12 (SF-12) Physical Component Score (SF-12 PCS) and Mental Component Score (SF-12 MHS).

Higher total MSPSS scores were correlated with better patient-reported outcomes, including lower levels of fatigue (*r*=0.21, *P* = 0.008), pain (*r*=−0.17, *P* = 0.03), depressive symptoms (*r*=−0.26, *P* < 0.001), and anxiety (*r*=−0.23, *P* = 0.004), along with better sleep quality (*r*=−0.32, *P* < 0.001) and higher SF-12 MCS scores (*r*=0.26, *P* < 0.001; Figure 3 and Supplemental Table 2). In investigating the MSPSS subscale associations, higher friend support demonstrated similar associations with these patient-reported outcomes (Supplemental Table 2). Higher family support evidenced similar significant associations as the total MSPSS score, with the exception of depressive symptoms (Supplemental Table 2). Higher significant other support demonstrated fewer significant associations with patient-reported outcomes (Supplemental Table 2). Higher family, friend, significant other, and total MSPSS support scores were all associated with higher SF-12 MCS (*r*=0.20, *P* = 0.010; *r*=0.24, *P* = 0.002; *r*=0.20, *P* = 0.01; and *r*=0.26, *P* < 0.001, respectively); and higher MSPSS friend support was associated with higher SF-12 PCS (*r*=0.16, *P* = 0.04; Supplemental Table 2). Associations between similar PROMIS subscales (anxiety, depression, fatigue, pain, physical function, and sleep interference) and MSPSS total and subscale scores were of the same direction but different magnitudes (Supplemental Table 2). Not surprisingly, higher total MSPSS scores and higher friend support were significantly associated with higher scores on the social roles subscale (*r*=−0.26, *P* < 0.001; *r*=−0.35, *P* < 0.001, respectively).

**Figure 4:**
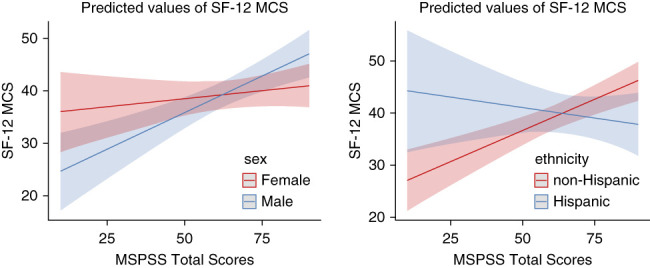
**Association between total MSPSS and SF-12 MCS stratified by sex/ethnicity.** MCS, Mental Component Score.

Using linear regression, after adjusting for age, sex, race, ethnicity, and CCI, MSPSS total scores were significantly associated with SF-12 MCS (*β*=0.17, *P* = 0.001; Table 2). Similar results were found using stepwise adjusted analysis for friends, family, and significant other social support predicting SF-12 MCS (Supplemental Table 3). By contrast, MSPSS total scores were not associated with SF-12 PCS after adjusting for these covariates (Table 2). Moderation analyses of MSPSS total scores on SF-12 MCS revealed two significant interactions, sex (*β*=−13.58; 95% CI, −26.09 to −1.07; *P* = 0.03) and ethnicity (*β*=−0.32; 95% CI, −0.55 to −0.09; *P* = 0.007), such that when sex was male or ethnicity was non-Hispanic, the estimated effect of MSPSS total on SF-12 MCS was significantly greater (Figure 4 and Supplemental Table 4).

**Table 2:** Multivariable linear regression analyses—outcome Short Form-12 Physical Component Score and Mental Component Score

Dependent and Independent Variables	Model 0		Model 1		Model 2	
	*β* Coefficient (95% CI)	*P* Value	*β* Coefficient (95% CI)	*P* Value	*β* Coefficient (95% CI)	*P* Value
**SF-12 PCS**						
MSPSS total	0.06 (−0.03 to 0.15)	0.218	0.07 (−0.02 to 0.16)	0.136	0.07 (−0.02 to 0.17)	0.103
Age			−0.06 (−0.16 to 0.03)	0.195	−0.02 (−0.12 to 0.08)	0.645
Male			2.96 (0.35 to 5.57)	0.026	3.15 (0.58 to 5.72)	0.016
Black			−3.04 (−7.41 to 1.33)	0.171	−3.03 (−7.32 to 1.26)	0.165
White			−4.23 (−8.43 to −0.04)	0.048	−4.27 (−8.40 to −0.14)	0.043
Other race			−5.32 (−12.04 to 1.39)	0.120	−5.74 (−12.35 to 0.87)	0.088
Hispanic			−0.32 (−4.10 to 3.45)	0.866	−1.06 (−4.82 to 2.69)	0.577
CCI					−0.98 (−1.74 to −0.21)	0.013
**SF-12 MCS**						
MSPSS total	0.16 (0.07 to 0.26)	<0.001	0.17 (0.07 to 0.26)	<0.001	0.17 (0.07 to 0.26)	<0.001
Age			0.12 (0.02 to 0.22)	0.018	0.10 (−0.01 to 0.20)	0.067
Male			0.33 (−2.36 to 3.02)	0.809	0.22 (−2.47 to 2.91)	0.872
Black			−4.37 (−8.88 to 0.14)	0.058	−4.37 (−8.87 to 0.13)	0.057
White			−4.51 (−8.85 to −0.18)	0.041	−4.49 (−8.81 to −0.17)	0.042
Other race			−5.30 (−12.23 to 1.64)	0.133	−5.06 (−11.98 to 1.87)	0.151
Hispanic			−0.44 (−4.34 to 3.46)	0.825	−0.01 (−3.95 to 3.92)	0.994
CCI					0.56 (−0.25 to 1.36)	0.173

Model 0: Multidimensional Scale of Perceived Social Support total. CCI, Charlson Comorbidity Index; CI, confidence interval; MCS, Mental Component Score; MSPSS, Multidimensional Scale of Perceived Social Support; PCS, Physical Component Score; SF-12, Short Form-12.

Model 1: Model 0+age, sex, race, ethnicity.

Model 2: Model 1+Charlson Comorbidity Index.

## Discussion

Perceived social support in this sample was comparable with that of other chronically ill populations, was associated with two sociodemographic variables (marital status and education) but no disease-related variables, and was associated with a range of patient-reported outcomes. Perceived social support among the patients enrolled in the TACcare trial were moderate and comparable with other hemodialysis samples^11,16,19^ and chronically ill populations, including those with heart failure, chronic obstructive pulmonary disease, diabetes mellitus, and Parkinson disease.^20^ Support from friends was slightly lower than support from family and significant others, similar to another study with patients receiving hemodialysis^8^ and a study with other chronically ill populations,^20^ suggesting that social networks were relatively limited for these patients receiving hemodialysis. Time-intensive treatment and related side effects, such as fatigue, may make it difficult to have social relations outside of the immediate family.^1–4^

To address whether sociodemographic and disease-related characteristics affect perceived social support of patients receiving hemodialysis, being married and having high school education were associated with higher levels of perceived social support, but no disease-related characteristics were associated with total MSPSS scores. One study of Turkish patients receiving hemodialysis found that marital status was not associated with perceived social support,^10^ but in a sample of American older adults, being married was associated with greater perceived social support from a significant other and from family, measured by the MSPSS, than being unmarried.^30^ Importantly, the marriage rate in our sample (27%) was significantly lower than the US marriage rate,^31^ suggesting that ESKD may affect the access to or durability of marriage. Education level has not been evaluated in relation to MSPSS scores among patients receiving hemodialysis, but in a study with patients receiving hemodialysis from Vietnam and with another social support measure, higher education was associated with better social support, perhaps because those with higher education have more time and resources to engage in social interactions.^32^ The limited literature addressing the association between disease-related characteristics and social support among patients receiving hemodialysis is mixed, with findings in both directions and with samples outside of the United States.^12,16,33^

There was a significant association between perceived social support and patient-reported outcomes; higher total MSPSS scores were correlated with a range of better patient-reported outcomes, including lower levels of fatigue, pain, depressive symptoms, and anxiety, as well as better sleep quality and higher SF-12 MCSs. This aligns with other studies evidencing significant associations between social support and patient-reported outcomes,^9–13,33–35^ including SF-12 MCSs.^32^ Of the three sources of support, higher friend support evidenced the highest correlation with mental well-being (SF-12 MCS), followed by family and significant other support. Importantly, higher family, friend, significant other, and total MSPSS support scores were all associated with higher mental well-being, and higher perceived friend support was associated with higher physical well-being (SF-12 PCS). Perceived friend support was found to be more strongly related to HRQoL domains than other support forms in a study of patients receiving hemodialysis from Korea.^10^ In this way, friend support may play a particularly important role in the lives of patients receiving hemodialysis with often depleted friendship networks.

Associations between PROMIS subscales (anxiety, depression, fatigue, pain, physical function, and sleep interference) and MSPSS total and subscale scores were in the same direction but were of different magnitudes, providing additional validation of findings. Not surprisingly, higher total MSPSS scores and higher perceived friend support were associated with higher scores on the social roles subscale of the SF-12.

After adjusting for age, sex, race, ethnicity, and CCI, perceived social support was still associated with better mental well-being (SF-12 MCS). By contrast, MSPSS total scores were *not* statistically associated with better physical well-being (SF-12 PCS) after adjusting for these covariates. Thus, the relationship between perceived social support and the more psychosocial dimensions of patient-reported outcomes, including social functioning, role limitations due to emotional problems, and mental health, appears to be particularly robust among patients receiving hemodialysis. Supporting this finding, a study examining types of functional social support (tangible, emotional/informational, positive social interaction, and affection), as opposed to sources of support, found that greater levels of social support independently and positively predicted mental health, but not physical health, and mitigated the effects of depression on physical health.^32^

Importantly, ESKD has been associated with, on average, moderate levels of loneliness, with the greatest level of loneliness reported in the positive social interaction domain.^36^ Patients receiving hemodialysis with high levels of loneliness reported more depressive symptoms, greater family dysfunction, and less social support.^36^ More generally, among patients with other chronic diseases, such as chronic lymphocytic leukemia, social support has been found to moderate the relationship between physical symptom burden and psychological symptoms.^37^

There were important factors moderating the relationship between perceived social support and patient-reported outcomes. The moderation analyses of perceived social support and mental well-being (SF-12 MCS) revealed two significant interactions, sex and ethnicity, such that perceived social support had stronger positive associations with mental well-being among male and non-Hispanic participants. Women on hemodialysis have been found to demonstrate poorer psychosocial well-being, including lower scores on mental and physical well-being,^33^ but their associations with social support were unknown. Social support is, in part, culturally constructed. How individuals conceptualize social support and what they expect from others may vary by culture,^15^ with some preliminary evidence that social support may have culturally based differential associations among patients receiving hemodialysis.^16^

Because of the associations between perceived social support and patient-reported outcomes, particularly mental health, targeting social support appears to be warranted among patients receiving hemodialysis. The findings of this study should be interpreted in light of several important limitations. The cohort was from a randomized controlled trial that screened patients into the study if they reported high levels of depressive symptoms, pain, and fatigue and, therefore, may have limited generalizability. Furthermore, we do not have data on whether these symptoms were present before hemodialysis or began after hemodialysis initiation, thereby limiting our understanding of the role of hemodialysis in precipitating and/or exacerbating symptoms. However, the observed characteristics of the study participants reflected the demographics of the study sites, and the dialysis characteristics were similar to the general ESKD population. 11.3% of patients with ESKD assessed for study eligibility were ineligible because of their limited English proficiency, thereby limiting our findings' generalizability to non–English-speaking patients with ESKD. The study used self-reported social support; however, the MSPSS was collected by interviewers yielding a high degree of data capture. Finally, we did not take into account the social role of the dialysis unit as a social network where lonely people can interact positively with caregivers and other patients, as suggested in a recent AKI Rehabilitation intervention study.^38^

This study demonstrated that social support was limited among patients undergoing in-center dialysis. Social support is a significant modifiable risk factor and improved social support may positively affect morbidity and mortality of patients receiving hemodialysis through a variety of hypothesized mechanisms, including lower depressive symptoms, improved HRQoL, increased access to health care, better adherence to medical treatments, and direct effects on the immune system.^7^ In fact, among chronically ill populations, social support has been shown to improve depressive symptoms, the most common psychological symptom among patients with ESKD, directly through the provision of emotional or functional support and indirectly through the perception of decreased isolation and loneliness.^9,14^ Improved depressive symptoms, in turn, enhance perceptions of quality of life among these patients.^9,14^ Future ESKD studies should evaluate how the social environment and networks in a dialysis unit can be leveraged to enhance perceived social support and its subsequent effect on HRQoL and symptom burden.

Health care professionals should, therefore, pay particular attention to patients' support networks and intervene to improve them. Future longitudinal studies are warranted to investigate the associations between social support and patient-reported outcomes over time. The multidisciplinary care team in the hemodialysis clinical platform provides an opportunity to apply these study findings by monitoring social support through the work of social workers and the medical team. In future work, interventions to enhance social support could be tested among patients receiving hemodialysis to improve patient-reported outcomes.

## Supplementary Material

## Data Availability

All data is included in the manuscript and/or supporting information.

## Supplemental Material

Supplemental Table 1. Comparison of means scores on MSPSS with respect to sociodemographic and disease-related variables.

Supplemental Table 2. Correlations between health-related quality of life outcomes and perceived social support.

Supplemental Table 3. Stepwise adjusted analysis for friends, family, and significant other social support predicting SF-12 MCS.

Supplemental Table 4. Moderation analysis of MSPSS total on SF-12 MCS.

## References

[1] Abdel-Kader K Unruh ML Weisbord SD. Symptom burden, depression, and quality of life in chronic and end-stage kidney disease. Clin J Am Soc Nephrol. 2009;4(6):1057–1064. doi:10.2215/CJN.00430109 19423570 PMC2689883

[2] Davison SN Koncicki H Brennan F. Pain in chronic kidney disease: a scoping review. Semin Dial. 2014;27(2):188–204. doi:10.1111/sdi.12196 24517512

[3] Weisbord SD Fried LF Arnold RM, . Prevalence, severity, and importance of physical and emotional symptoms in chronic hemodialysis patients. J Am Soc Nephrol. 2005;16(8):2487–2494. doi:10.1681/ASN.2005020157 15975996

[4] Fletcher BR Damery S Aiyegbusi OL, . Symptom burden and health-related quality of life in chronic kidney disease: a global systematic review and meta-analysis. PLoS Med. 2022;19(4):e1003954. doi:10.1371/journal.pmed.1003954 35385471 PMC8985967

[5] Centers for Disease Control and Prevention. Measuring Healthy Days: Population Assessment of Health-Related Quality of Life. Centers for Disease Control and Prevention; 2000.

[6] O’Hare AM Richards C Szarka J, . Emotional impact of illness and care on patients with advanced kidney disease. Clin J Am Soc Nephrol. 2018;13(7):1022–1029. doi:10.2215/CJN.14261217 29954826 PMC6032592

[7] Cohen SD Sharma T Acquaviva K Peterson RA Patel SS Kimmel PL. Social support and chronic kidney disease: an update. Adv Chronic Kidney Dis. 2007;14(4):335–344. doi:10.1053/j.ackd.2007.04.007 17904500

[8] Mollaoglu M. Perceived social support, anxiety and self care among patients receiving hemodialysis. Dial Transplant. 2006;35(3):144–155. doi:10.1002/dat.20002

[9] Patel SS Peterson RA Kimmel PL. The impact of social support on end stage renal disease. Semin Dial. 2005;18(2):98–102. doi:10.1111/j.1525-139X.2005.18203.x 15771652

[10] Karadag E Kilic SP Metin O. Relationship between fatigue and social support in hemodialysis patients. Nurs Health Sci. 2013;15(2):164–171. doi:10.1111/nhs.12008 23552015

[11] Kang GW Lee IH Ahn KS Lee J Ji Y Woo J. Clinical and psychosocial factors predicting health-related quality of life in hemodialysis patients. Hemodial Int. 2015;19(3):439–446. doi:10.1111/hdi.12271 25643587

[12] Kim K Kang GW Woo J. The quality of life of hemodialysis patients is affected not only by medical but also psychosocial factors: a canonical correlation study. J Korean Med Sci. 2018;33(14):e111. doi:10.3346/jkms.2018.33.e111 29607636 PMC5879041

[13] Lilympaki I Makri A Vlantousi K Koutelekos I Babatsikou F Polikandrioti M. Effect of perceived social support on the levels of anxiety and depression of hemodialysis patients. Mater Sociomed. 2016;28(5):361–365. doi:10.5455/msm.2016.28.361-365 27999485 PMC5149439

[14] Cohen SD. Social support interventions will improve the quality of life of ESRD patients. Semin Dial. 2013;26(3):262–265. doi:10.1111/sdi.12064 23432395

[15] American Psychological Association. Multicultural Guidelines: An Ecological Approach to Context, Identity, and Intersectionality. American Psychological Association; 2017.

[16] Yarlioglu M OguzGundogmus EA Atiglan K Sahin H Ayli M. The relationship between depression, anxiety, quality of life levels, and the chronic kidney disease stage in the autosomal dominant polycystic kidney disease. Inter Urol Nephrol. 2023;55(4):983–992. doi:10.1007/s11255-022-03375-2 36184721

[17] Jhamb M Steel J Yabes J, . Effects of technology assisted stepped collaborative care intervention to improve symptoms in patients undergoing hemodialysis: the TĀCcare randomized clinical trial. JAMA Intern Med. 2023;183(8):795–805. doi:10.1001/jamainternmed.2023.2215 37338898 PMC10282960

[18] Zimet GD Powell SS Farley GK Werkman S Berkoff KA. Psychometric characteristics of the multidimensional scale of perceived social support. J Personal Assess. 1990;55(3-4):610–617. doi:10.1080/00223891.1990.9674095 2280326

[19] Kimmel PL Peterson RA Weihs KL, . Psychosocial factors, behavioral compliance and survival in urban hemodialysis patients. Kidney Int. 1998;54(1):245–254. doi:10.1046/j.1523-1755.1998.00989.x 9648085

[20] De Maria M Vellone E Durante A Biagioli V Matarese M. Psychometric evaluation of the multidimensional scale of perceived social support (MSPSS) in people with chronic diseases. Ann Ist Super Sanita. 2018;54(4):308–315. doi:10.4415/ANN_18_04_07 30575567

[21] Cella D Wilson H Shalhoub H, . Content validity and psychometric evaluation of Functional Assessment of Chronic Illness Therapy-Fatigue in patients with psoriatic arthritis. J Patient Rep Outcomes. 2019;3(1):30. doi:10.1186/s41687-019-0115-4 31111255 PMC6527714

[22] Upadhyay C Cameron K Murphy L Battistella M. Measuring pain in patients undergoing hemodialysis: a review of pain assessment tools. Clin Kidney J. 2014;7(4):367–372. doi:10.1093/ckj/sfu067 25852910 PMC4377812

[23] Beck AT Steer RA Brown G. Beck Depression Inventory–II (BDI-II) [Database Record]. APA PsycTests; 2006. doi:10.1037/t00742-000.

[24] Buysse DJ Reynolds CF III Monk TH Berman SR Kupfer DJ. The Pittsburgh Sleep Quality Index: a new instrument for psychiatric practice and research. Psychiatry Res. 1989;28(2):193–213. doi:10.1016/0165-1781(89)90047-4 2748771

[25] Lowe B Decker O Muller S, . Validation and standardization of the generalized anxiety disorder screener (GAD-7) in the general population. Med Care. 2008;46(3):266–274. doi:10.1097/MLR.0b013e318160d093 18388841

[26] Washburn RA McAuley E Katula J Mihalko SL Boileau RA. The physical activity scale for the elderly (PASE): evidence for validity. J Clin Epidemiol. 1999;52(7):643–651. doi:10.1016/s0895-4356(99)00049-9 10391658

[27] Rand Health Care. 12-Item Short Form Survey (SF-12). 1995. Accessed July 16, 2022. https://www.rand.org/health-care/surveys_tools/mos/12-item-short-form.html

[28] Cella D Riley W Stone A, . The Patient Reported Outcomes Measurement Information System (PROMIS) developed and tested its first wave of adult selfreported health outcome item banks: 2005-2008. J Clin Epidemiol. 2010;63(11):1179–1194. doi:10.1016/j.jclinepi.2010.04.011 20685078 PMC2965562

[29] R Core Team. R: A Language and Environment for Statistical Computing. R Foundation for Statistical Computing; 2023. Accessed November 16, 2023. https://www.R-project.org/

[30] Stanley M Beck J Zebb J. Psychometric properties of the MSPSS in older adults. Aging Ment Health. 1998;2(3):186–193. doi:10.1080/13607869856669

[31] Kaplan RM Kronick RG. Marital status and longevity in the United States population. J Epidemiol Community Health. 2006;60(9):760–765. doi:10.1136/jech.2005.037606 16905719 PMC2566023

[32] Hoang VL Green T Bonner A. Examining social support, psychological status and health-related quality of life in people receiving haemodialysis. J Ren Care. 2022;48(2):102–111. doi:10.1111/jorc.12380 34041850

[33] Simms RJ Thong KM Dworschak GC Ong AC. Increased psychosocial risk, depression and reduced quality of life living with autosomal dominant polycystic kidney disease. Nephrol Dial Transplant. 2016;31(7):1130–1140. doi:10.1093/ndt/gfv299 26268712

[34] Chan R Steel Z Brooks R, . Psychosocial risk and protective factors for depression in the dialysis population: a systematic review and meta-regression analysis. J Psychosom Res. 2011;71(5):300–310. doi:10.1016/j.jpsychores.2011.05.002 21999973

[35] Christensen AJ Ehlers SL. Psychological factors in end- stage renal disease: an emerging context for behavioral medicine research. J Consult Clin Psychol. 2002;70(3):712–724. doi:10.1037//0022-006x.70.3.712 12090378

[36] Pallone JM Santos DGMD Oliveira Dias AL Ferreira LG Costa da Silva C Orlandi FdS. Loneliness level and its associated factors in patients with hemodialysis. Clin Nurs Res. 2022;31(6):1164–1171. doi:10.1177/10547738211061447 34955033

[37] Morrison EJ Flynn JM Jones J Byrd JC Andersen BL. Individual differences in physical symptom burden in individuals with chronic lymphocytic leukemia. Ann Hematol. 2016;95(12):1989–1997. doi:10.1007/s00277-016-2790-z. 27539615 PMC9097783

[38] Singh G Hu Y Jacobs S, . Post-discharge mortality and rehospitalization among participants in a comprehensive Acute kidney Injury rehabilitation program. Kidney360. 2021;2(9):1424–1433. doi:10.34067/KID.0003672021 35373103 PMC8786140

