# Supplementary material for: Associations between Social Support and Patient-Reported Outcomes in Patients Receiving Hemodialysis: Results from the TACcare Study
Source: Kidney360. 2024 May 3;5(6):860–9. doi: 10.34067/KID.0000000000000456 (PMC11219113; doi:10.34067/KID.0000000000000456)
Supplement: Supplementary file 1 [file kidney360-5-860-s001.pdf]

ASN American Society of Nephrology | JASN Journal of the American Society of Nephrology | KIDNEY360 Accessing Our World From Every Angle | CJASN Clinical Journal of the American Society of Nephrology

ASN Journal Disclosure Form

As per ASN journal policy, I have disclosed any financial relationship or commitment held by myself and/or my spouse/partner in the past 36 months as included below. I have listed my Current Employer below to indicate there is a relationship requiring disclosure. If no relationship exists, my Current Employer is not listed.

S. Erickson reports the following:
Employer: UNM

I understand that the information above will be published within the journal article, if accepted, and that failure to comply and/or to accurately and completely report the potential financial conflicts of interest could lead to the following: 1) Prior to publication, article rejection, or 2) Post-publication, sanctions ranging from, but not limited to, issuing a correction, reporting the inaccurate information to the authors' institution, banning authors from submitting work to ASN journals for varying lengths of time, and/or retraction of the published work.

Name: Sarah Jane Erickson
Manuscript ID: K360-2023-000876R1
Manuscript Title: Associations between social support and patient-reported outcomes in hemodialysis patients: Results from the TACcare Study
Date of Completion: January 22, 2024
Disclosure Updated Date: May 17, 2023

ASN Journal Disclosure Form

As per ASN journal policy, I have disclosed any financial relationship or commitment held by myself and/or my spouse/partner in the past 36 months as included below. I have listed my Current Employer below to indicate there is a relationship requiring disclosure. If no relationship exists, my Current Employer is not listed.

Z. Han has nothing to disclose.

I understand that the information above will be published within the journal article, if accepted, and that failure to comply and/or to accurately and completely report the potential financial conflicts of interest could lead to the following: 1) Prior to publication, article rejection, or 2) Post-publication, sanctions ranging from, but not limited to, issuing a correction, reporting the inaccurate information to the authors' institution, banning authors from submitting work to ASN journals for varying lengths of time, and/or retraction of the published work.

Name: Zhuoheng Han
Manuscript ID: K360-2023-000876R1
Manuscript Title: Associations between social support and patient-reported outcomes in hemodialysis patients: Results from the TACcare Study
Date of Completion: January 23, 2024
Disclosure Updated Date: May 17, 2023

ASN American Society of Nephrology | JASN Journal of the American Society of Nephrology | KIDNEY360 Accessing Our World From Every Angle | CJASN Clinical Journal of the American Society of Nephrology

ASN Journal Disclosure Form

As per ASN journal policy, I have disclosed any financial relationship or commitment held by myself and/or my spouse/partner in the past 36 months as included below. I have listed my Current Employer below to indicate there is a relationship requiring disclosure. If no relationship exists, my Current Employer is not listed.

M. Jhamb reports the following:
Employer: University of Pittsburgh and University of Pittsburgh Medical Center; Consultancy: Xcenda, LLC; Boehringer Ingelheim LLC, Networks of Excellence, Eli-Lilly; Research Funding: NIH, Dialysis Clinic, Inc., Bayer LLC, Pfizer; and Other Interests or Relationships: Member of ASN and National Kidney Foundation.

I understand that the information above will be published within the journal article, if accepted, and that failure to comply and/or to accurately and completely report the potential financial conflicts of interest could lead to the following: 1) Prior to publication, article rejection, or 2) Post-publication, sanctions ranging from, but not limited to, issuing a correction, reporting the inaccurate information to the authors' institution, banning authors from submitting work to ASN journals for varying lengths of time, and/or retraction of the published work.

Name: Manisha Jhamb
Manuscript ID: K360-2023-000876R1
Manuscript Title: Associations between social support and patient-reported outcomes in hemodialysis patients: Results from the TACcare Study
Date of Completion: January 29, 2024
Disclosure Updated Date: January 29, 2024

ASN American Society of Nephrology | JASN Journal of the American Society of Nephrology | KIDNEY360 Accessing Our World From Every Angle | CJASN Clinical Journal of the American Society of Nephrology

ASN Journal Disclosure Form

As per ASN journal policy, I have disclosed any financial relationship or commitment held by myself and/or my spouse/partner in the past 36 months as included below. I have listed my Current Employer below to indicate there is a relationship requiring disclosure. If no relationship exists, my Current Employer is not listed.

B. Rollman reports the following:
Employer: University of Pittsburgh School of Medicine

I understand that the information above will be published within the journal article, if accepted, and that failure to comply and/or to accurately and completely report the potential financial conflicts of interest could lead to the following: 1) Prior to publication, article rejection, or 2) Post-publication, sanctions ranging from, but not limited to, issuing a correction, reporting the inaccurate information to the authors' institution, banning authors from submitting work to ASN journals for varying lengths of time, and/or retraction of the published work.

Name: Bruce L. Rollman
Manuscript ID: K360-2023-000876R1
Manuscript Title: Associations between social support and patient-reported outcomes in hemodialysis patients: Results from the TACcare Study
Date of Completion: March 4, 2024
Disclosure Updated Date: March 4, 2024

ASN American Society of Nephrology | JASN Journal of the American Society of Nephrology | KIDNEY360 Accessing Our World From Every Angle | CJASN Clinical Journal of the American Society of Nephrology

ASN Journal Disclosure Form

As per ASN journal policy, I have disclosed any financial relationship or commitment held by myself and/or my spouse/partner in the past 36 months as included below. I have listed my Current Employer below to indicate there is a relationship requiring disclosure. If no relationship exists, my Current Employer is not listed.

M. Roumelioti reports the following:
Employer: University of New Mexico; Consultancy: My spouse: Baxter, Otsuka, Bayer, Quanta, Integrity; and Other Interests or Relationships: Participating in DCI quality meetings and receiving financial support.

I understand that the information above will be published within the journal article, if accepted, and that failure to comply and/or to accurately and completely report the potential financial conflicts of interest could lead to the following: 1) Prior to publication, article rejection, or 2) Post-publication, sanctions ranging from, but not limited to, issuing a correction, reporting the inaccurate information to the authors' institution, banning authors from submitting work to ASN journals for varying lengths of time, and/or retraction of the published work.

Name: Maria-Eleni Roumelioti
Manuscript ID: K360-2023-000876R1
Manuscript Title: Associations between social support and patient-reported outcomes in hemodialysis patients: Results from the TACcare Study
Date of Completion: January 29, 2024
Disclosure Updated Date: June 18, 2023

ASN Journal Disclosure Form

As per ASN journal policy, I have disclosed any financial relationship or commitment held by myself and/or my spouse/partner in the past 36 months as included below. I have listed my Current Employer below to indicate there is a relationship requiring disclosure. If no relationship exists, my Current Employer is not listed.

J. Steel reports the following:
Employer: University of Pittsburgh; and Patents or Royalties: Springer.

I understand that the information above will be published within the journal article, if accepted, and that failure to comply and/or to accurately and completely report the potential financial conflicts of interest could lead to the following: 1) Prior to publication, article rejection, or 2) Post-publication, sanctions ranging from, but not limited to, issuing a correction, reporting the inaccurate information to the authors' institution, banning authors from submitting work to ASN journals for varying lengths of time, and/or retraction of the published work.

Name: Jennifer L. Steel
Manuscript ID: K360-2023-000876R1
Manuscript Title: Associations between social support and patient-reported outcomes in hemodialysis patients: Results from the TACcare Study
Date of Completion: March 4, 2024
Disclosure Updated Date: March 4, 2024

ASN
American Society of Nephrology
JASN
Journal of the American Society of Nephrology
KIDNEY360
Accessing Our World From Every Angle
CJASN
Clinical Journal of the American Society of Nephrology

ASN Journal Disclosure Form

As per ASN journal policy, I have disclosed any financial relationship or commitment held by myself and/or my spouse/partner in the past 36 months as included below. I have listed my Current Employer below to indicate there is a relationship requiring disclosure. If no relationship exists, my Current Employer is not listed.

M. Unruh reports the following:
Employer: University of New Mexico

I understand that the information above will be published within the journal article, if accepted, and that failure to comply and/or to accurately and completely report the potential financial conflicts of interest could lead to the following: 1) Prior to publication, article rejection, or 2) Post-publication, sanctions ranging from, but not limited to, issuing a correction, reporting the inaccurate information to the authors' institution, banning authors from submitting work to ASN journals for varying lengths of time, and/or retraction of the published work.

Name: Mark L. Unruh
Manuscript ID: K360-2023-000876R1
Manuscript Title: Associations between social support and patient-reported outcomes in hemodialysis patients: Results from the TACcare Study
Date of Completion: January 29, 2024
Disclosure Updated Date: January 29, 2024

ASN American Society of Nephrology | JASN Journal of the American Society of Nephrology | KIDNEY360 Accessing Our World From Every Angle | CJASN Clinical Journal of the American Society of Nephrology

ASN Journal Disclosure Form

As per ASN journal policy, I have disclosed any financial relationship or commitment held by myself and/or my spouse/partner in the past 36 months as included below. I have listed my Current Employer below to indicate there is a relationship requiring disclosure. If no relationship exists, my Current Employer is not listed.

S. Weisbord reports the following:
Employer: University of Pittsburgh Medical Center; and Consultancy: Takeda.

I understand that the information above will be published within the journal article, if accepted, and that failure to comply and/or to accurately and completely report the potential financial conflicts of interest could lead to the following: 1) Prior to publication, article rejection, or 2) Post-publication, sanctions ranging from, but not limited to, issuing a correction, reporting the inaccurate information to the authors' institution, banning authors from submitting work to ASN journals for varying lengths of time, and/or retraction of the published work.

Name: Steven D. Weisbord
Manuscript ID: K360-2023-000876R1
Manuscript Title: Associations between social support and patient-reported outcomes in hemodialysis patients: Results from the TACcare Study
Date of Completion: February 1, 2024
Disclosure Updated Date: February 1, 2024

ASN Journal Disclosure Form

As per ASN journal policy, I have disclosed any financial relationship or commitment held by myself and/or my spouse/partner in the past 36 months as included below. I have listed my Current Employer below to indicate there is a relationship requiring disclosure. If no relationship exists, my Current Employer is not listed.

J. Yabes reports the following:
Employer: University of Pittsburgh; and Research Funding: Bayer.

I understand that the information above will be published within the journal article, if accepted, and that failure to comply and/or to accurately and completely report the potential financial conflicts of interest could lead to the following: 1) Prior to publication, article rejection, or 2) Post-publication, sanctions ranging from, but not limited to, issuing a correction, reporting the inaccurate information to the authors' institution, banning authors from submitting work to ASN journals for varying lengths of time, and/or retraction of the published work.

Name: Jonathan Yabes
Manuscript ID: K360-2023-000876R1
Manuscript Title: Associations between social support and patient-reported outcomes in hemodialysis patients: Results from the TACcare Study
Date of Completion: January 24, 2024
Disclosure Updated Date: May 19, 2023
