# Supplementary material for: Associations between Social Support and Patient-Reported Outcomes in Patients Receiving Hemodialysis: Results from the TACcare Study
Source: Kidney360. 2024 May 3;5(6):860–9. doi: 10.34067/KID.0000000000000456 (PMC11219113; doi:10.34067/KID.0000000000000456)
Supplement: Supplementary file 2 [file kidney360-5-860-s002.pdf]

Supplementary Table 1. Comparison of MSPSS Scores with Respect to Sociodemographic and Disease-related Variables

| Sociodemographic Characteristic | Significant Other Support Mean (SD) | Friend Support Mean (SD) | Family Support Mean (SD) | Total Support Mean (SD) |
|---|---|---|---|---|
| **Sex** | | | | |
| Female | 22.31 (5.73) | 19.39 (6.45) | 21.56 (5.39) | 63.25 (15.12) |
| Male | 21.69 (4.88) | 20.12 (5.80) | 21.06 (5.57) | 62.88 (13.03) |
| **Marital Status** | | | | |
| Married | **23.91 (3.85)** | 19.93 (6.80) | **22.95 (4.17)** | **66.79 (12.68)** |
| Not Married | **21.26 (5.55)** | 19.74 (5.84) | **20.67 (5.78)** | **61.67 (14.21)** |
| **Age** | | | | |
| < 65 years | 22.19 (5.51) | 19.64 (6.38) | 21.08 (5.89) | 62.90 (14.89) |
| > 65 years | 21.45 (4.68) | 20.17 (5.38) | 21.77 (4.34) | 63.38 (11.58) |
| **Race** | | | | |
| American Indian | 21.95 (6.17) | 17.90 (7.56) | 21.38 (6.49) | 61.24 (16.91) |
| Black | 21.57 (5.17) | 19.33 (6.08) | 20.70 (5.30) | 61.59 (13.75) |
| White | 22.14 (5.06) | 20.45 (5.69) | 21.47 (5.26) | 64.06 (13.16) |
| Missing/Other | 22.40 (6.20) | 20.50 (5.91) | 22.20 (6.36) | 65.10 (15.90) |
| **Ethnicity** | | | | |
| Hispanic | 22.71 (5.59) | 19.93 (6.97) | 21.93 (5.76) | 64.57 (16.14) |
| Non-Hispanic | 21.81 (5.21) | 19.77 (5.92) | 21.14 (5.43) | 62.72 (13.51) |
| **Education Level** | | | | |
| < high school | 22.47 (5.17) | 16.58 (7.77) | 18.84 (6.78) | **57.89 (13.85)** |
| >= high school | 21.90 (5.30) | 20.23 (5.73) | 21.61 (5.22) | **63.74 (13.88)** |
| **Employment** | | | | |
| Employed | 21.44 (5.17) | 20.11 (3.72) | 19.00 (4.77) | 60.56 (11.30) |
| Not employed | 22.21 (5.67) | 19.62 (6.63) | 21.11 (5.99) | 62.94 (15.89) |
| Retired | 21.76 (4.86) | 19.96 (5.73) | 21.77 (4.89) | 63.49 (11.87) |
| **Income** | | | | |
| < $40K | 22.89 (5.26) | 19.92 (6.28) | 22.32 (4.19) | 65.13 (13.44) |
| > $40K | 21.68 (5.27) | 19.75 (6.06) | 20.96 (5.79) | 62.39 (14.12) |
| **SDI** | | | | |
| <= median | 21.66 (5.50) | 19.51 (6.14) | 20.89 (5.41) | 62.06 (13.66) |
| > median | 22.17 (5.04) | 20.00 (6.11) | 21.72 (5.57) | 63.89 (14.42) |
| **Rural-urban** | | | | |
| Urban | 21.84 (5.29) | 19.62 (6.15) | 21.20 (5.55) | 62.66 (14.07) |
| Rural | 23.86 (4.60) | 23.00 (3.96) | 23.86 (2.48) | 70.71 (10.37) |
| **Walkability Score** | | | | |
| Car-dependent | **22.53 (5.20)** | 20.02 (6.10) | 21.65 (5.36) | 64.21 (13.62) |
| Walkable | **21.00 (5.29)** | 19.41 (6.11) | 20.64 (5.66) | 61.05 (14.43) |

| **Disease-related Characteristic** | **Significant Other Support Mean (SD)** | **Friend Support Mean (SD)** | **Family Support Mean (SD)** | **Total Support Mean (SD)** |
|---|---|---|---|---|
| **Diabetes** | | | | |
| Yes | 21.97 (5.10) | 19.94 (6.39) | 21.40 (5.58) | 63.31 (13.98) |
| No | 21.97 (5.60) | 19.54 (5.59) | 21.08 (5.34) | 62.59 (14.04) |
| **CVD** | | | | |
| Yes | 21.51 (5.20) | 19.93 (5.80) | 20.67 (5.44) | 62.10 (13.58) |
| No | 22.30 (5.33) | 19.70 (6.33) | 21.72 (5.49) | 63.72 (14.27) |
| **Etiology of ESKD** | | | | |
| Diabetic nephropathy | 22.29 (5.17) | 20.32 (6.48) | 22.21 (5.15) | 64.82 (13.76) |
| Hypertensive nephrosclerosis | 20.92 (5.78) | 18.42 (5.64) | 19.46 (5.09) | 58.81 (14.05) |
| Other | 22.00 (5.20) | 19.70 (5.72) | 20.84 (5.90) | 62.54 (13.99) |
| **Dialysis Vintage** | | | | |
| < 18 months | 22.19 (5.24) | 19.65 (6.54) | 21.12 (6.46) | 62.96 (15.28) |
| 18-32 months | 22.71 (4.31) | 20.11 (6.66) | 21.68 (5.24) | 64.50 (12.82) |
| > 32 months | 21.60 (5.60) | 19.77 (5.69) | 21.24 (4.98) | 62.61 (13.67) |

*The bold font of mean (SD) indicates there is significant difference (p-value < 0.05) between the characteristic

MSPSS: Multi-dimensional Scale of Perceived Social Support, SDI: State disability insurance, CVD: Cardiovascular disease, ESKD: End Stage Kidney Disease.

Supplementary Table 2. Correlations Between Health-related Quality of Life Outcomes and Perceived Social Support

| Characteristics | MSPSS Sig Other Correlation (p-value) | MSPSS Friends Correlation (p-value) | MSPSS Family Correlation (p-value) | MSPSS Total Correlation (p-value) |
|---|---|---|---|---|
| **FACIT-F** | 0.081 (0.309) | 0.284 (<0.001) | 0.140 (0.078) | 0.209 (0.008) |
| **PROMIS Fatigue** | -0.071 (0.376) | -0.211 (0.008) | -0.167 (0.035) | -0.184 (0.020) |
| **BPI Pain Score** | -0.063 (0.431) | -0.162 (0.040) | -0.186 (0.018) | -0.167 (0.034) |
| **PROMIS Pain Interference** | -0.101 (0.206) | -0.295 (<0.001) | -0.183 (0.021) | -0.239 (0.002) |
| **BDI** | -0.156 (0.048) | -0.309 (<0.001) | -0.166 (0.036) | -0.259 (<0.001) |
| **PROMIS Depression** | -0.220 (0.005) | -0.277 (<0.001) | -0.220 (0.005) | -0.291 (<0.001) |
| **PSQI Sleep Score** | -0.222 (0.005) | -0.340 (<0.001) | -0.226 (0.004) | -0.320 (<0.001) |
| **PROMIS Sleep Disturbance** | -0.092 (0.248) | -0.157 (0.049) | -0.050 (0.529) | -0.123 (0.012) |
| **GAD-7** | -0.103 (0.195) | -0.296 (<0.001) | -0.146 (0.065) | -0.225 (0.004) |
| **PROMIS Anxiety** | -0.064 (0.422) | -0.184 (0.020) | -0.128 (0.106) | -0.155 (0.051) |
| **PASE Physical Activity** | 0.129 (0.105) | 0.091 (0.251) | 0.092 (0.249) | 0.124 (0.117) |
| **PROMIS Physical Function** | -0.017 (0.829) | -0.150 (0.059) | 0.044 (0.578) | -0.054 (0.495) |
| **PROMIS Social Roles** | -0.151 (0.058) | -0.352 (<0.001) | -0.135 (0.091) | -0.263 (<0.001) |
| **SF-12 PCS** | 0.013 (0.868) | 0.161 (0.042) | 0.059 (0.461) | 0.098 (0.218) |
| **SF-12 MCS** | 0.199 (0.012) | 0.245 (0.002) | 0.203 (0.010) | 0.261 (<0.001) |

MSPSS: Multi-dimensional Scale of Perceived Social Support, FACIT-F: Functional Assessment of Chronic Illness Therapy Fatigue, PROMIS: NIH Patient Reported Outcomes Measurement Information System, BPI: Brief Pain Inventory-Short Form, BDI: Beck Depression Inventory, PSQI: Pittsburgh Sleep Quality Index, GAD-7: Generalized Anxiety Disorder-7, PASE: Physical Activity Scale for Elderly, Medical Outcomes Study Short Form-12 (SF-12) Physical Component Score (SF-12 PCS) and Mental Component Score (SF-12 MHS).

Supplementary Table 3. Stepwise Adjusted Analysis for Friends, Family, and Significant Other Social Support Predicting SF-12 MCS

| Covariates | Estimate (95% CI) of Model 0 | P-value | Estimate (95% CI) of Model 1 | P-value | Estimate (95% CI) of Model 2 | P-value |
|---|---|---|---|---|---|---|
| **MSPSS Family** | 0.32 (0.08, 0.57) | 0.010 | 0.32 (0.07, 0.56) | 0.011 | 0.31 (0.07, 0.26) | 0.014 |
| **Age** | | | 0.12 (0.01, 0.22) | 0.027 | 0.09 (-0.01, 0.20) | 0.094 |
| **Male** | | | 0.37 (-2.38, 3.11) | 0.793 | 0.25 (-2.47, 2.91) | 0.857 |
| **Black** | | | -4.08 (-8.68, 0.52) | 0.082 | -4.09 (-8.87, 0.13) | 0.080 |
| **White** | | | -4.03 (-8.44, 0.38) | 0.073 | -4.02 (-8.81, -0.17) | 0.073 |
| **Other Race** | | | -4.86 (-11.92, 2.20) | 0.176 | -4.62 (-11.98, 1.87) | 0.197 |
| **Hispanic** | | | -0.53 (-4.51, 3.44) | 0.791 | -0.09 (-3.95, 3.92) | 0.965 |
| **CCI** | | | | | 0.58 (-0.25, 1.36) | 0.162 |

| Covariates | Estimate (95% CI) of Model 0 | P-value | Estimate (95% CI) of Model 1 | P-value | Estimate (95% CI) of Model 2 | P-value |
|---|---|---|---|---|---|---|
| **MSPSS Friends** | 0.35 (0.13, 0.57) | 0.002 | 0.37 (0.15, 0.59) | 0.001 | 0.37 (0.07, 0.26) | 0.001 |
| **Age** | | | 0.12 (0.02, 0.22) | 0.022 | 0.09 (-0.01, 0.20) | 0.085 |
| **Male** | | | 0.04 (-2.67, 2.74) | 0.979 | -0.08 (-2.47, 2.91) | 0.952 |
| **Black** | | | -4.65 (-9.18, -0.11) | 0.045 | -4.65 (-8.87, 0.13) | 0.044 |
| **White** | | | -4.84 (-9.21, -0.46) | 0.030 | -4.82 (-8.81, -0.17) | 0.030 |
| **Other Race** | | | -5.51 (-12.49, 1.47) | 0.121 | -5.2 (-11.98, 1.87) | 0.138 |
| **Hispanic** | | | -0.15 (-4.06, 3.77) | 0.941 | 0.32 (-3.95, 3.92) | 0.874 |
| **CCI** | | | | | 0.62 (-0.25, 1.36) | 0.133 |

| Covariates | Estimate (95% CI) of Model 0 | P-value | Estimate (95% CI) of Model 1 | P-value | Estimate (95% CI) of Model 2 | P-value |
|---|---|---|---|---|---|---|
| **MSPSS Significant Others** | 0.33 (0.07, 0.59) | 0.012 | 0.36 (0.11, 0.62) | 0.005 | 0.35 (0.10, 0.61) | 0.007 |
| **Age** | | | 0.13 (0.03, 0.23) | 0.011 | 0.11 (0.00, 0.22) | 0.044 |
| **Male** | | | 0.46 (-2.27, 3.20) | 0.738 | 0.35 (-2.38, 3.09) | 0.800 |
| **Black** | | | -4.31 (-8.88, 0.27) | 0.065 | -4.31 (-8.87, 0.25) | 0.064 |
| **White** | | | -4.23 (-8.62, 0.16) | 0.059 | -4.21 (-8.59, 0.17) | 0.060 |
| **Other Race** | | | -4.92 (-11.94, 2.11) | 0.169 | -4.69 (-11.70, 2.33) | 0.189 |
| **Hispanic** | | | -0.52 (-4.47, 3.44) | 0.796 | -0.10 (-4.10, 3.89) | 0.959 |
| **CCI** | | | | | 0.54 (-0.28, 1.36) | 0.196 |

MSPSS: Multi-dimensional Scale of Perceived Social Support, CCI: Charlson Comorbidity Index

Supplementary Table 4. Moderation Analysis of MSPSS Total on SF-12 MCS

| **Covariates** | **Interaction Term Estimate (95% CI)** | **P-value** |
|---|---|---|
| **Age** | 0.00 (-0.01, 0.01) | 0.630 |
| **Male** | 0.22 (0.02, 0.41) | 0.027 |
| **Black** | -0.09 (-0.38, 0.20) | 0.533 |
| **White** | -0.10 (-0.36, 0.17) | 0.466 |
| **Other Race** | 0.01 (-0.41, 0.42) | 0.974 |
| **Hispanic** | -0.32 (-0.55, -0.09) | 0.007 |
